# The Role of Daytime Sleepiness in Psychosocial Outcomes after Treatment for Obstructive Sleep Apnea

**DOI:** 10.1155/2013/140725

**Published:** 2013-03-31

**Authors:** Esther Yuet Ying Lau, Gail A. Eskes, Debra L. Morrison, Malgorzata Rajda, Kathleen F. Spurr

**Affiliations:** ^1^Sleep Laboratory, Department of Psychology, 6/F Jockey Club Tower, The University of Hong Kong, Pokfulam Road, Hong Kong; ^2^Department of Psychiatry, Dalhousie University, Halifax, NS, Canada; ^3^Department of Psychology, Dalhousie University, Halifax, NS, Canada; ^4^Department of Medicine, Dalhousie University, Halifax, NS, Canada; ^5^Sleep Clinic and Laboratory, Queen Elizabeth II Health Sciences Centre, Halifax, NS, Canada; ^6^School of Health Sciences, Dalhousie University, Halifax, NS, Canada

## Abstract

We investigated the role of daytime sleepiness and sleep quality in psychosocial outcomes of patients with obstructive sleep apnea (OSA) treated with continuous positive airway pressure (CPAP). Thirty-seven individuals with moderate to severe OSA and compliant with CPAP treatment for at least 3 months were compared to 27 age- and education-matched healthy controls. The OSA group and the control group were studied with overnight polysomnography (PSG) and compared on measures of daytime sleepiness (Epworth Sleepiness Scale), sleep quality (Pittsburg Sleep Quality Index), mood (Beck Depression Inventory, Profile of Mood States), and functional outcomes (Functional Outcomes of Sleep Questionnaire). After CPAP treatment, the OSA group improved on sleep quality and sleepiness. As a group, they did not differ from controls on sleep architecture after CPAP. The OSA group also showed significant improvements in functional outcomes and was comparable to controls on mood and functional outcomes. Persistent difficulties included lowered activity level and residual sleepiness in some individuals. Sleepiness was found to be a significant predictor of mood and affective states, while both sleepiness and sleep quality predicted functional outcomes. These results highlight the importance of assessment and intervention targeting psychosocial functioning and sleepiness in individuals with OSA after treatment.

## 1. Introduction

Individuals with obstructive sleep apnea-hypopnea syndrome (OSA) experience excessive daytime sleepiness and fatigue, decreased cognitive function and mood changes, resulting in significant, negative consequences in work and driving performance, and lowered quality of life (see review by [1]). Therefore, the evaluation of OSA treatment on both nighttime and daytime consequences of OSA is critical.

The most obvious consequence and manifestation of untreated OSA are probably subjective sleepiness and high propensity to fall asleep during the daytime. Engleman and Douglas [2] reviewed 29 studies that measured sleepiness and concluded that at least moderate impairments in terms of excessive daytime sleepiness are indicated in patients with OSA. Accumulating evidence suggests that the main causes of daytime sleepiness in patients with OSA are sleep fragmentation and sleep architecture disruptions [3]. Some propose that sleepiness of patients with more severe OSA may be more related to the breathing disruptions and the associated nocturnal hypoxemia (e.g., [4]).

An association between OSA and mood disorders is revealed by studies reporting their comorbidity (e.g., [5]). Previous studies also showed that individuals with OSA showed elevated scores on measures of depression, and 58% met the DSM criteria for depression [6]. In the Wisconsin Sleep Cohort Study, longitudinal data demonstrated a dose-response association between OSA and depression in a community sample of 1408 participants [7]. On the contrary, other studies do not find an association between OSA and psychological problems (e.g., [8]). Authors like Cassel contend that the so-called personality change or psychological consequence of OSA is due to a misinterpretation of sleepiness by medical staff and the overlap of symptoms like fatigue between OSA and depression. The implication is that symptoms of fatigue and depression have to be distinguished in studies investigating mood in patients with OSA. Bardwell et al. [9] concluded from their study that many of the previously reported links between mood and OSA dissipate after controlling for covariates such as age, BMI, and hypertension. But it should be noted that a strong relationship between OSA and mood was demonstrated even after potential confounds were controlled in a later study (see above) [5].

The relationship between OSA and psychological problems is still uncertain, and the mechanisms of OSA-related mood symptoms are unknown. Depressive symptoms in patients with OSA have been proposed to be related to oxygen desaturation and nocturnal hypoxemia (e.g., [10]) or to sleep fragmentation and excessive daytime sleepiness [11]. It has also been shown that experimental sleep fragmentation produces significant mood symptoms [12], supporting the potential effects of sleep disruption and daytime sleepiness on mood. However, it is difficult, if not impossible, for previous studies to distinguish the more direct effects of the illness itself (i.e., the hypoxic insults) and the indirect effects of daytime sleepiness on mood in patients with untreated OSA. Investigating stably treated individuals in which hypoxemia is eliminated or minimized would help elucidate the remaining effects of any persistent daytime sleepiness. 

Continuous Positive Airway Pressure (CPAP) has been reported to be effective in improving subjective and objective measures of sleepiness in patients with OSA [13], with therapeutic effects ranging from moderate to large [2, 14]. The average improvement for patients with severe OSA was 4.75 points on the Epworth Sleepiness Scale (ESS, [13]). However, it is not uncommon for individual patients to continue to experience excessive daytime sleepiness with CPAP treatment (e.g., [4]). A number of authors have reported improvements of psychological functioning after CPAP treatment (e.g., [15]). Several placebo-controlled studies on the reversibility of psychopathology (mainly depression) have been conducted with mixed results [16, 17]. Sateia [18] concluded that while the clinical impression suggests that OSA may be directly related to psychopathology and that treatments can reverse the impairments, the literature does not provide unequivocal support for these associations. In addition, another question that is left unanswered is whether the psychosocial functioning of stably treated patients is comparable to their peers without OSA. 

The purpose of the current study was to investigate the relationship between any residual sleepiness or sleep issues and psychosocial functioning in individuals with stably treated OSA to understand their long-term outcomes. In order to ensure that the patients were indeed properly treated with CPAP, data on their pre-treatment status (i.e., PSG data, sleepiness scores, subjective sleep quality) were collected and compared with posttreatment data. Improvements on hypoxemia indices, sleepiness, and sleep quality were previously reported in an earlier paper focusing on cognitive functions [19] and will be summarized in the results section. Here, we report in detail on how changes in sleepiness and sleep quality relate to psychosocial functioning posttreatment. The OSA group was also asked to rate their pre-treatment functional outcomes retrospectively to understand if and to what extent they perceived CPAP helped in terms of subjective daytime functions. We then investigated the predictors of the psychosocial functioning of individuals with treated OSA. Outcome variables included the mood measures and functional outcomes. Predictors examined were demographics, pre-treatment disease severity, and posttreatment sleep-related variables.

Based on the findings of previous studies, we hypothesized that (1) the OSA group will show improvements in their daytime sleepiness, sleep quality, and functional outcomes after CPAP treatment as compared to their pre-CPAP status; (2) current sleep variables would be more important in predicting psychosocial outcomes in individuals with stably treated OSA compared to pre-treatment diagnostic variables; (3) the associations between sleepiness, sleep quality, and psychosocial functioning would also apply to healthy age-matched controls. 

## 2. Methods

### 2.1. Participants and Procedures

Thirty-seven individuals with OSA treated with CPAP and 27 healthy control participants were studied. Details of the inclusion and exclusion criteria were reported in Lau et al. [19]. Briefly, patients had moderate to severe OSA and had been on CPAP treatment for at least three months with compliance of at least 4 hours per night for 80% of the week; exclusion criteria were other sleep pathologies as verified by overnight PSG and clinical interview by attending physicians, diagnosed psychiatric disorders (e.g., psychotic disorders, major depression with suicidal ideation), or neurological conditions (e.g., stroke, epilepsy), current alcohol or drug abuse, current use of medication that could affect cognitive function (e.g., benzodiazepine), and undertreatment of OSA other than CPAP. The protocol was approved by the Research Ethics Board of the Queen Elizabeth II Health Sciences Centre and was prepared in accordance with the Helsinki Declaration. After the initial screening and consenting procedure, all participants filled out questionnaires of subjective sleep quality, sleepiness, and psychosocial measures (see below). Then, participants completed experimental tasks of working memory and a comprehensive neuropsychological battery (findings reported in Lau et al. [19]). All participants also received an overnight PSG using standardized procedures and scoring within eight weeks of psychological testing [20]. Relevant information was extracted from patients' medical charts at the Sleep Clinic and Laboratory, including pre-treatment data on PSG, daytime sleepiness, sleep quality, and other relevant medical history and medications.

### 2.2. Measures

#### 2.2.1. Epworth Sleepiness Scale (ESS) [21, 22]

All participants completed the ESS, a self-administered eight-item questionnaire for measuring daytime sleepiness by rating the chances of dozing off or falling asleep in eight different situations commonly encountered in daily life, on a scale from 0 (would never doze) to 3 (high chance of dozing). An ESS score > 10 (out of 24) is conventionally considered as clinically significant [23]. ESS is a validated clinical and research tool in assessing daytime sleepiness with high test-retest reliability (*r* = 0.82) and internal consistency (Cronbach's alpha = 0.88) [21, 24, 25]. The reliable change index of 5.89 on the ESS as calculated by a formula with the mean change score, the standard deviation, and the reliability coefficient are used in the current study to evaluate the extent of change in our OSA group [26]. 

#### 2.2.2. Pittsburgh Sleep Quality Index (PSQI)

The PSQI is a self-rated questionnaire which assesses sleep quality and disturbances over a one-month time interval. Nineteen individual items generate seven “component” scores including subjective sleep quality, sleep latency, sleep duration, habitual sleep efficiency, sleep disturbances, use of sleeping medication, and daytime dysfunction. Component scores ranging from 0 to 3 are then summed to yield one global score (0–21) with higher scores indicating worse sleep quality. The global score is used to distinguish good and poor sleepers (>5 as poor sleepers) with a diagnostic sensitivity of 89.6% and specificity of 86.5%. Evidence of internal homogeneity (Cronbach's *α* = 0.83) and consistency (test-retest reliability coefficient = 0.85) was reported by Buysse et al. [27]. 

#### 2.2.3. Beck Depression Inventory (BDI) [28]

On the well-validated BDI, mild, moderate, and severe levels of depressive symptomatology are indicated by a raw score of 14 to 19, 20 to 28, and 29 to 63, respectively. 

#### 2.2.4. Profile of Mood States (POMS) [29]

Affective states were measured by the POMS. Participants rate their feelings during the past week on 65 adjective scales using a 5-point rating system from 0 (not at all) to 4 (extremely). The profile includes a total score and scores of six affective states: tension anxiety, depression dejection, anger hostility, vigor activity, fatigue inertia, and confusion bewilderment. These subscales separate out somatic symptoms (sleepiness, fatigue) from affective symptoms (anxiety, depression), providing useful evidence to validate the potential effectiveness of CPAP in improving mood, without the confound of physical symptoms. The reliability and validity of the POMS have been demonstrated in numerous studies in a variety of normal as well as chronically ill populations [29–31]. 

#### 2.2.5. Functional Outcomes of Sleep Questionnaire (FOSQ) [32]

The FOSQ was used to evaluate the specific impact of excessive sleepiness or tiredness on multiple activities of everyday living. A total score can be generated from five factors including activity level, vigilance, intimacy, sexual relationships, general productivity, and social outcome. The FOSQ has been found to be capable of discriminating between normal participants and untreated sleep apnea patients [32]. It has very high content validity, test-retest reliability (*r* = 0.91), and internal consistency (Cronbach's alpha = 0.96). A total score of less than 18 is considered as clinically significant [23]. Participants were asked to fill out the questionnaires according to their current condition and then their condition before using CPAP. This is the “Then-Test” approach designed to eliminate treatment-induced response-shift effects and used to provide an unconfounded indication of treatment effects [33]. 

### 2.3. Data Analytical Strategies

Significant improvements in PSG variables, daytime sleepiness, and subjective sleep quality were reported in Lau et al. [19]. In the current study, we first assessed the posttreatment changes in functional outcomes in the OSA group using paired sample *t*-test. To understand the clinical significance of the changes, the percentages of patients and healthy controls whose scores were in the clinical range also were compared using chi-square analyses. Between-group independent *t*-tests were used to compare the scores of the OSA group and healthy controls on sleepiness (ESS), sleep quality (PSQI), and on psychosocial outcomes, namely, mood (BDI, POMS) and daytime functioning (FOSQ). 

To explore the predictors of patients' psychosocial outcomes, mood measures (BDI, POMS), and subjective daytime functioning (FOSQ) were regressed on predictors, including age, BMI, diagnostic RDI, diagnostic minimum SpO_2_, posttreatment sleep efficiency, current sleepiness (ESS), and subjective sleep quality (PSQI) using stepwise regression procedures. 

Due to the number of analyses, a significance level of .01 was used for all between-group comparisons to control for Type I error. Significance level for regression analyses was set at .05 due to their exploratory nature.

## 3. Results

### 3.1. Participants' Characteristics

Participants' characteristics and the OSA group's clinical information were reported by Lau et al. [19] and summarized in Table 1. Briefly, the two groups were of comparable age and education level but as expected, the mean BMI of the OSA group was significantly higher than that of the control group, and the gender ratio differed between the two groups with more males in the OSA group and more females in the control group. 

### 3.2. Effects of CPAP Treatment on Nighttime Sleep, Daytime Sleepiness, and Functional Outcomes

**Figure 1:**
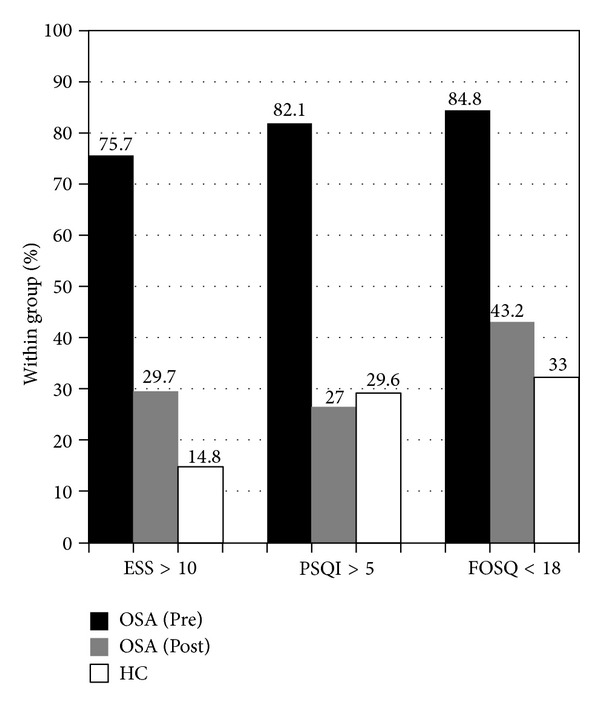
Percentages of participants with OSA pre- and posttreatment and of controls scoring in the clinically significant range on the Epworth Sleepiness Scale (ESS), Pittsburg Sleep Quality Index (PSQI—global score), and Functional Outcomes of Sleep Questionnaire (FOSQ—total score).

Significant improvements on respiratory and oxygen saturation indices (RDI, minimum SpO_2_, and mean SpO_2_), sleepiness (ESS), and sleep quality (PSQI) were reported by Lau et al. [19] and briefly reported in Table 1. There were also significant posttreatment improvements in functional outcomes (FOSQ), *t* = 7.64 (32), *P* < .000, and *d* = 0.91. For measures that have established clinical cut-offs, the percentage of individuals of the OSA group and that of the control group passing the thresholds for the clinically problematic range are shown in Figure 1. Overall, there were large reductions in the percentages of individuals with OSA in the clinically significant range on ESS, PSQI, and FOSQ after CPAP treatment. However, 30% of the posttreatment OSA group still reported excessive daytime sleepiness, twice the percentage in the healthy control group. Also, 43% of OSA group (posttreatment) had significant problems in functional outcomes, as compared to 33% of the controls. These differences in the percentages of clinically significant scores between the two groups were not statistically significant using chi-square analyses, however.

### 3.3. Comparisons between Treated OSA Group and Healthy Controls on Sleep and Psychosocial Outcomes

Results of the between-group comparisons are summarized in Table 2. There was a trend for a difference between the two groups in the component score of daytime dysfunction on the PSQI, *t*(62) = 2.14, *P* = .036, with the OSA group reporting more sleep-related daytime dysfunction than the controls did. 

No significant differences between the two groups were detected on the two mood measures, BDI and POMS. On the BDI, 5% of patients (versus none in the control group) showed an elevated score in the mild range (14–19). In the FOSQ, the only difference found was on the FOSQ subscale of “activity level,” with the OSA group having a lower activity level than controls, *t*(62) = 3.00, *P* < .01. 

### 3.4. Predictors of Psychosocial Outcomes in Treated OSA Group

#### 3.4.1. Emotional Functioning

BDI was predicted by posttreatment ESS score, with sleepier patients having a higher level of depressive symptoms (Table 3). Posttreatment ESS also predicted POMS-total score, with worse affective states associated with sleepiness. To delineate the affective versus the physical components underlying mood states, individual subscales of POMS were regressed on the same set of variables (Table 4). Posttreatment ESS predicted all subscales except for vigor activity, which was predicted by PSQI, with poorer sleep quality associated with lower vigor activity. In addition to ESS, BMI also predicted fatigue inertia, with higher BMI associated with worse fatigue inertia. To investigate whether these associations apply also to the control group, post hoc analyses on the correlations between ESS scores with BDI and POMS were conducted. Significant correlation between ESS and POMS was found in the control group (total score—*r* = .57, *P* < .01, tension anxiety—*r* = .40, *P* < .05, depression dejection—*r* = .53, *P* < .01, fatigue inertia—*r* = .69, *P* < .001). 

#### 3.4.2. Functional Outcomes

 Posttreatment FOSQ—total score was predicted by posttreatment ESS score and posttreatment PSQI—global score. Better functional outcomes were associated with less sleepiness and better sleep quality (Table 5). FOSQ—activity level was associated with lower scores on the ESS, lower scores on the PSQI, and older age. FOSQ—vigilance was associated with lower ESS scores. FOSQ—intimate relationships and sexual activity, general productivity, and social outcomes were all associated with lower PSQI scores. These associations were not found in the control group. 

## 4. Discussion

This study aimed at elucidating the role of daytime sleepiness in psychosocial outcomes of individuals with OSA treated with CPAP. Pre- and posttreatment comparisons on subjective sleep quality (PSQI), daytime sleepiness (ESS), and functional outcomes (FOSQ) revealed significant improvements, consistent with findings of some previous studies [13, 15, 34]. 

### 4.1. Is Daytime Sleepiness an Issue in Stably Treated Individuals with OSA?

The percentage of individuals in the OSA group with self-reports of pathological sleepiness dropped from 76% to 30% after treatment, with a significant mean change of 6.1 (c.f. the reliable change index score of 5.9 [26] and consistent with the mean reduction of 4.75 points as reported by a comprehensive review [35]). It could be concluded that sleepiness was significantly reduced in the OSA group after CPAP treatment, although one-third of individuals continued to show excessive sleepiness during the day (compared to 15% of controls). Given that sleepiness was found to consistently predict psychosocial outcomes in this study, the question of what predicts residual sleepiness in treated individuals with OSA is relevant. No significant predictors of posttreatment ESS scores were identified in post hoc analyses. We also investigated the potential causes as suggested by Santamaria et al. [35], including inadequate CPAP treatment or titration, insufficient sleep, depression, or other sleep disorders. Issues with compliance or titration were unlikely to be the culprit as our study participants had good compliance and titration level was deemed suitable as validated by PSG. In addition, mean hours of CPAP usage were not correlated with ESS scores (*r* = −.259, *P* = .122). With regards to sleep restriction, ESS scores were also not correlated with sleep duration in either the OSA group or the control group. Other sleep pathologies were ruled out by PSG as well. There was an association between ESS scores and BDI scores, but it could not be concluded that the residual sleepiness was related to depression as the mean BDI score was well within the normal range (details discussed below). With the lack of statistical difference in self-reported sleepiness between the two groups, one could argue that the sleepiness in the OSA group could just be a normal age-related phenomenon. To investigate the potential aging effects on sleepiness, the percentage of OSA group with an ESS score above 2 standard deviations of the mean of the control group was calculated. 45.9% and 5.4% of the OSA group (pre- and posttreatment) were found to have ESS scores above this age-corrected cut-off (16/17). These percentages are much less than those generated with the original cut-off score, which was based on a group of 72 healthy individuals in the age range of 22 to 59 [36]. Taken together, the sleepiness as shown in the OSA group posttreatment may not be much more than what one may expect from a healthy person in his/her 50s. Nevertheless, given the predictive power of posttreatment ESS on psychosocial outcomes in the OSA group found in this study, in addition to the rich literature on the associations between daytime somnolence and various health variables such as high blood pressure, obesity, coronary artery disease, and cerebrovascular disease [37, 38], which are very common in individuals with OSA, the issue of excessive sleepiness undoubtedly deserves more attention in this medically vulnerable group of patients. Optimizing treatment for individual patients whose excessive daytime sleepiness persists with compliant CPAP usage focusing on strategies for reducing and managing sleepiness is needed. 

### 4.2. Are Sleep Quality, Functional Outcomes, and Psychosocial Outcomes Problems for Stably Treated OSA Individuals?

A similar pattern of findings was shown in self-reported sleep quality, as the proportion of poor sleepers fell from 82% before treatment to 27% after treatment in the OSA group, as compared to 30% in the control group. As patients and controls did not differ on the PSQI (except for the trend for the OSA group having worse daytime dysfunction, which essentially taps into sleepiness), and the two groups were mostly comparable on PSG measures, it could be concluded that sleep quality of the OSA group was largely normalized. 

No significant differences between the two groups were detected on the two mood measures, BDI and POMS. The percentages of people with clinical level of depressive symptoms were also comparable between individuals with OSA treated with CPAP and controls. These findings are in contrast with those reported by Vernet et al. in their recent study [4] as their healthy controls and treated OSA groups with and without residual sleepiness had higher BDI scores than our participants, ranging from 20 to 70% having elevated scores (adopted cut-off: >12), as compared to 0% and 8% in our control and OSA groups, respectively. These differences suggest that variability in psychosocial functioning across different patient samples may be noteworthy even when inclusion/exclusion criteria are similar. In view of the gender ratio difference between the two groups, BDI and POMS scores were regressed on gender in order to investigate the potential contribution of gender on mood or symptom reporting. Gender did not predict any mood measures (*P* > .05). 

There was a trend toward individuals with OSA to have a reduced energy level even after treatment with CPAP, seen on subscales from both the POMS and the FOSQ. Thus, individuals with OSA seemed to continue to have a compromised level of energy and activity level after CPAP treatment. While exercise programs implemented in patients with untreated OSA have been found to be effective in reducing fatigue and depressive symptoms [39], such interventions should also be considered for OSA-treated individuals with reduced energy level. 

### 4.3. Predictors of Psychosocial Outcomes in Treated OSA Group

Our data showed that self-reported sleepiness was a potent predictor of mood and functional outcomes, while subjective sleep quality predicted functional outcomes. Age was found to be a significant predictor of activity level, while BMI was associated with fatigue. 

#### 4.3.1. Emotional Functioning

While the mean level of depressive symptoms (2.8) endorsed by patients on the BDI was well within the minimal range (i.e., 0 to 13) and the percentage of patients with BDI scores higher than the clinical cut-off was only 5%, our data showed that patients' mood was still associated with their daytime sleepiness. Our findings did not directly address the question regarding the association of OSA and mood as there was no pre-treatment mood measure. Nevertheless, the finding that mood was predicted by sleepiness suggests a relationship between daytime sleepiness associated with OSA and mood. This relationship is in keeping with the Wisconsin Sleep Cohort Study [7], which demonstrated a causal link between OSA and depression, although the current study does not address the directionality of the relationship. Significant correlations between ESS and POMS scores in the control group highlight the importance of daytime sleepiness in emotional functioning in a broad sense, above and beyond the OSA illness. 

Several studies reported a lack of correlation between OSA and psychological functioning [40–42]. Some authors argue that sleepiness and fatigue are misinterpreted as depression [8], and yet others suggest that links between mood and OSA dissipate after controlling for covariates such as age, BMI, and hypertension in the analyses [9]. In this study, age and BMI were not significant predictors of BDI scores, suggesting that the association of sleepiness and mood is independent of these factors. In regard to the argument that sleepiness and fatigue in patients with OSA are misinterpreted as mood symptoms, our findings on the POMS may offer some insight. The total score and subscale scores separate out somatic symptoms (vigor, fatigue) from affective symptoms (anxiety, depression). We found that sleepiness (ESS) was associated with five of the six subscales, namely, tension anxiety, depression dejection, anger hostility, fatigue inertia, and confusion bewilderment, and, interestingly, not with vigor activity. The relationships with all the affective subscales, but not a physical scale (vigor activity), may suggest that the mood symptoms and their associations with sleepiness in patients with OSA are separable from the manifestations of fatigue. 

#### 4.3.2. Functional Outcomes

Better functional outcomes were associated with less sleepiness and better subjective sleep quality; specifically, sleepiness predicted activity level and vigilance, while sleep quality predicted intimate relationships and sexual activity, general productivity, social outcome, and activity level. These findings concur with the finding that sleep quality is an independent predictor of daytime fatigue in untreated OSA [43]. Taken together and consistent with the findings on mood, current sleepiness and sleep quality play a crucial role in daytime functioning of individuals generally classified as “well treated.” These relationships are not found in the healthy control group, despite the fact that the two groups seem to have comparable sleep quality, suggesting that the impact of the same level of quality of sleep may be different for individuals with and without a history of OSA and that there may be some specific factors that make an individual with OSA more susceptible to the adverse effects of poor sleep even after their OSA is well controlled by CPAP.

## 5. Conclusions

Previous studies of psychosocial functioning of individuals with OSA usually adopt one instrument to study one aspect of emotional functioning. We included multiple instruments for measuring mood and functional outcomes to more fully understand how OSA impacts the daily living of an individual who is compliant to the standard treatment of CPAP. Predictors of posttreatment psychosocial outcomes have not been adequately studied, and this study offers some preliminary evidence for future studies to build on; even though our sample size was only moderate, not many predictors could be included. Other caveats of this study were the baseline differences between gender ratio and the BMI of the two groups, although gender did not predict any outcomes and BMI was only associated with fatigue. 

As found in this study, daytime sleepiness and subjective sleep quality are important predictors of functional outcomes and mood. It is pivotal to explore specific strategies targeting such factors [44]. For example, it would be valuable to study how interventions like sleep hygiene education and lifestyle management (e.g., controlling tobacco and alcohol use, weight control) affect sleep quality and daytime sleepiness and the respective impact on daytime functioning and emotional functioning. As most of these interventions as well as the CPAP usage itself involve drastic behavioral changes and adaptations, motivational interviewing techniques could be a valuable component in inducing and maintaining changes in OSA patients with persistent problems in sleep and psychosocial functioning [45]. 

## Figures and Tables

**Table 1:** Participants' characteristics and OSA group CPAP treatment information.

	OSA before CPAP (A) (*n* = 37)	OSA after CPAP (B) (*n* = 37)	Controls (C) (*n* = 27)	A versus B *t* (df)	B versus C *t* (df)
Age (years)		57.9 (9.5)^a^	56.7 (10.5)		0.48 (62)
Sex (F : M)		15 : 22	19 : 8		
Education (years)		15.1 (3.6)	15.7 (3.2)		0.61 (62)
Body mass index (BMI)		33.5 (7.4)	25.5 (5.0)		4.83 (62)***
Time since diagnosis of OSA (months)		25.6 (21.1)			
Duration of CPAP treatment (months)		17.8 (11.4)			
Usage of CPAP per week (hours)		51.4 (6.7)			
CPAP compliance^b^		96.1 (5.6)			
Respiratory disturbance index (RDI)	42.2 (24.9)	1.7 (1.5)	4.0 (3.4)	9.52 (32)***	3.25 (62)**
Minimum oxygen saturation (min SpO2)	80.2 (9.8)	90.3 (3.6)	88.6 (3.3)	5.61 (27)***	1.90 (60)
Mean oxygen saturation (mean SpO2)	93.7 (3.5)	95.7 (1.6)	95.7 (0.9)	3.17 (29)**	0.10 (62)
Epworth Sleepiness Scale (ESS)	14.4 (5.2)	8.3 (4.5)	6.6 (4.7)	7.52 (36)***	1.48 (62)
Pittsburg Sleep Quality Index—global score	8.5 (3.3)	4.4 (2.4)	4.6 (2.8)	5.85 (27)***	0.20 (62)

CPAP: continuous positive airway pressure.

^a^Mean (SD).

^b^CPAP compliance is defined as percentage of days with usage >4 hours in the last 3 months. Compliance was determined objectively by downloading data from the built-in smart card of the CPAP machines for 73% of the participants. Self-report of usage was used for the rest of the participants whose machines were not equipped with a smart card.

**P < .01, ***P < .001.

**Table 2:** Comparison between the OSA group treated with CPAP and the control group in sleep and mood questionnaires.

	OSA (*n* = 37)	Controls (*n* = 27)	*t* (df = 62)	*P *	*d *
Epworth sleepiness scale (ESS)	8.3 (4.5)^a^	6.6 (4.7)	1.48	.144	0.38
Pittsburg sleep quality index (PSQI)					
Global score	4.4 (2.4)	4.6 (2.8)	0.29	.776	0.08
Subjective sleep quality	0.7 (0.6)	0.8 (0.7)	0.64	.526	0.17
Sleep latency	0.4 (0.6)	0.7 (0.6)	1.87	.066	0.50
Sleep duration	0.6 (0.8)	0.6 (0.7)	0.15	.880	0
Habitual sleep efficiency	0.3 (0.7)	0.6 (0.9)	1.38	.173	0.43
Sleep disturbances	1.2 (0.5)	1.2 (0.5)	0.47	.641	0
Use of sleep medication	0.2 (0.6)	0.1 (0.5)	0.29	.771	0.17
Daytime dysfunction	0.9 (0.7)	0.6 (0.6)	2.14	.036*	0.43
Beck Depression Inventory (BDI)	2.8 (4.7)^a^	2.9 (2.4)	0.01	.989	0.02
Profile of Mood States (POMS)					
Global score	67.4 (24.0)	60.3 (18.2)	1.29	.204	0.30
Tension anxiety	6.1 (5.0)	5.0 (4.7)	0.89	.375	0.22
Depression dejection	5.0 (7.1)	5.2 (6.1)	0.11	.913	0.03
Anger hostility	4.7 (6.2)	3.7 (4.0)	0.74	.465	0.16
Vigor activity	17.5 (6.7)	19.0 (5.2)	0.96	.342	0.22
Fatigue inertia	7.4 (6.2)	4.7 (4.9)	1.89	.064	0.44
Confusion bewilderment	6.2 (4.6)	5.3 (4.5)	0.78	.436	0.20
Functional Outcomes of Sleep Questionnaire (FOSQ)					
Total score	17.6 (2.2)	18.4 (1.5)	1.61	.113	0.36
Activity level	3.3 (0.6)	3.7 (0.3)	2.99	.004**	0.67
Vigilance	3.4 (0.5)	3.5 (0.5)	0.96	.341	0.20
Intimacy	3.6 (0.6)	3.8 (0.5)	1.04	.304	0.33
General productivity	3.6 (0.5)	3.8 (0.3)	1.48	.143	0.40
Social outcome	3.7 (0.5)	3.7 (0.5)	0.06	.953	0

Higher scores indicate more difficulties in all questionnaires, except for FOSQ.

^a^Mean (SD); *P < .05; **P < .01.

**Table 3:** Prediction of psychosocial outcomes in participants with OSA treated with CPAP using stepwise regression.

Predictors	BDI	POMS	FOSQ
ESS (post)			
*β*	0.45	0.54	−0.42
Δ*R* ^2^	.20	.29	.29
*t* (df)	2.59 (27)	3.33 (27)	−2.67 (26)
*P *	.015	.002	0.013
PSQI (post)			
*β*			−.37
Δ*R* ^2^			0.13
*t* (df)			−2.36 (26)
*P *			.026
Model *R* ^2^	.20	.29	.42
Adjusted *R* ^2^	.17	.27	.37
*F* (df)	6.71 (1, 27)	11.12 (1, 27)	9.31 (2, 26)
*P*	.015	.002	.001

Nonsignificant predictor variables including age, education, diagnostic respiratory disturbance index (RDI), diagnostic peripheral oxygen saturation (SpO_2_), and post-treatment sleep efficiency are not shown in the table.

ESS: Epworth Sleepiness Scale; PSQI: Pittsburg Sleep Quality Index; BDI: Beck Depression Inventory; POMS: Profile of Mood States; FOSQ: Functional Outcomes of Sleep Questionnaire.

**Table 4:** Prediction of Profile of Mood States subscales in participants with OSA treated with CPAP using stepwise regression.

Predictors	Tension anxiety	Depression dejection	Anger hostility	Vigor activity	Fatigue inertia	Confusion bewilderment
BMI						
*β*					0.38	
Δ*R* ^2^					.14	
*t* (df)					3.06 (26)	
*P *					.005	
ESS (post)						
*β*	0.48	0.39	0.46		0.47	0.47
Δ*R* ^2^	.23	.16	.21		.27	0.22
*t* (df)	2.83 (27)	2.23 (27)	2.70 (27)		3.06 (26)	2.78 (27)
*P *	.009	.035	.012		.005	.010
PSQI (post)						
*β*				−0.58		
Δ*R* ^2^				.33	
*t* (df)				−3.66 (27)		
*P *				.001		
Model *R* ^2^	.23	.15	.22	.33	.41	.22
Adjusted *R* ^2^	.20	.12	.18	.31	.37	.19
*F* (df)	7.99 (1, 27)	4.95 (1, 27)	7.28 (1, 27)	13.39 (1, 27)	9.14 (2, 26)	7.70 (1,27)
*P *	.009	.035	.012	.001	.001	.010

Nonsignificant predictor variables including age, education, diagnostic respiratory disturbance index (RDI), diagnostic peripheral oxygen saturation (SpO_2_), and post-treatment sleep efficiency are not shown in the table.

BMI: body mass index; ESS: Epworth sleepiness scale; PSQI: Pittsburg sleep quality index.

**Table 5:** Prediction of Functional Outcomes of Sleep Questionnaire subscales in participants with OSA treated with CPAP using stepwise regression.

Predictors	Activity level	Vigilance	Intimate relationships	General productivity	Social outcomes
Age					
*β*	0.33				
Δ*R* ^2^	.12				
*t* (df)	2.31 (25)				
*P *	.030				
ESS (post)					
*β*	−0.032	−0.66			
Δ*R* ^2^	.09	.43			
*t* (df)	−2.12 (25)	−4.52 (25)	−2.30 (23)		
*P *	.044	.000	.031		
PSQI (post)					
*β*	−0.43			−0.40	−0.16
Δ*R* ^2^	.28			.16	0.37
*t* (df)	−2.86 (25)			−2.28 (27)	−3.95 (27)
*P *	.009			.031	.001
Model *R* ^2^	.49	.43	.19	.43	.37
Adjusted *R* ^2^	.43	.41	.15	.41	.34
*F* (df)	8.13 (3, 25)	20.41 (1, 27)	5.29 (1, 23)	20.41 (1, 27)	15.61 (1, 27)
*P *	.001	.000	.031	.000	.001

Non-significant predictor variables including education, diagnostic respiratory disturbance index (RDI), diagnostic peripheral oxygen saturation (SpO_2_), and post-treatment sleep-efficiency are not shown in the table.

ESS: Epworth sleepiness scale; PSQI: Pittsburg sleep quality index.
